# Reply to “letter to the editor: Measuring renal hemodynamics during exercise using Doppler ultrasound”

**DOI:** 10.14814/phy2.16018

**Published:** 2024-04-16

**Authors:** Shotaro Kawakami, Tetsuhiko Yasuno, Saki Kawakami, Ai Ito, Kanta Fujimi, Takuro Matsuda, Shihoko Nakashima, Kosuke Masutani, Yoshinari Uehara, Yasuki Higaki, Ryoma Michishita

**Affiliations:** ^1^ Faculty of Sports and Health Science Fukuoka University Fukuoka Japan; ^2^ The Fukuoka University Institute for Physical Activity Fukuoka Japan; ^3^ Center for Liberal Arts and Sciences, Faculty of Engineering Toyama Prefectural University Imizu Japan; ^4^ Division of Nephrology and Rheumatology, Department of Internal Medicine Fukuoka University School of Medicine Fukuoka Japan; ^5^ Department of Rehabilitation Fukuoka University Hospital Fukuoka Japan

To the editor: We thank the authors of the Letter to the Editor Christopher Chapman, Rachel Drew, John Halliwill, Christopher Minson, and Zachary Schlader for their interest in our study and for their comments (Chapman et al., 2024).

The traditional method used to assess renal blood flow is para‐aminohippuric clearance (Castenfors, 1967; Grimby, 1965). This method provides helpful information. However, it is invasive and requires blood and urine samples and unable to quickly evaluate renal blood flow. Therefore, we used ultrasound echo that can be performed noninvasively and is able to quickly evaluate renal blood flow, which place less strain on participants compared with the traditional method. In addition, ultrasound echo allows for changes in blood flow velocity, and cross‐sectional area to be assessed following exercise. Therefore, we performed several studies to determine the effects of exercise on renal blood flow (renal hemodynamics) using ultrasound echo (Kawakami et al., 2018; Kawakami et al., 2022; Kawakami et al., 2023; Kotoku et al., 2019).

First, we state that the assessment of renal hemodynamics using Doppler ultrasonography was performed immediately after exercise, not during exercise (Kawakami et al., 2024). At the beginning, we tried to assess renal hemodynamics using Doppler ultrasonography during exercise. However, As the kidneys move with respiration and body movement, breathing needs to pause when assessing the renal hemodynamics, this makes it difficult to assess renal hemodynamics with high accuracy during exercise. Therefore, the assessment of renal hemodynamics using Doppler ultrasonography was performed immediately after exercise rather than during exercise (Kawakami et al., 2018; Kawakami et al., 2022; Kawakami et al., 2023; Kawakami et al., 2024; Kotoku et al., 2019). Considering that, as authors point out (Chapman et al., 2024), renal vascular resistance returns to resting levels within 40 s after stopping exercise (Endo et al., 2008), the present data may not strictly reflect renal hemodynamics during exercise.

As described in the paper (section “2.5 The assessment of renal hemodynamics”) (Kawakami et al., 2024), to evaluate renal hemodynamics as soon as possible immediately after exercise, the probe position was determined in advance and the location of probe was marked. As illustrated in Figure 2a, the renal hemodynamics were assessed with the subject sitting on the bicycle ergometer immediately after exercise, with one technician (T. Yasuno) handling the probe and another technician (S. Kawakami) operating the device to ensure smooth and accurate assessment of renal hemodynamics. The technician placed the probe on the left lumbar region of the subject and drew the renal artery using color Doppler method. Subsequently, the technician expanded the measurement screen and moved the cursor to the origin of the renal artery and measured blood flow velocity (the mean blood flow rate was defined as the average of three Doppler waveforms) using the pulse Doppler method. And the device (Aplio 300; Toshiba Medical Systems) we are using has a screen recording function and we recorded the measurement screen during the measurement with the pulse Doppler method. Thus, measurement of cross‐sectional area and calculation of renal blood flow can be performed later. Therefore, it is sufficient to be able to measure blood flow velocity with the pulse Doppler method and it does not take much time (approximately 30–60 s) between the cessation of exercise and renal hemodynamic assessment. Moreover, the ultrasound echo (Aplio 300; Toshiba Medical Systems) we are using can play back the recorded measurement screen frame by frame, we further expanded the recorded measurement screen and determined the cross‐sectional area. As the authors point out, there were also occasions when the renal arteries were obscured. In case where the renal artery delineated by the color Doppler method was unclear, the color Doppler mode of the expanded renal artery was removed and the renal artery was delineated by the B‐mode, and the renal artery diameter was measured. In addition, we had previously attempted to assess the renal hemodynamics using other ultrasound echoes, which failed to delineate the renal arteries and calculate renal blood flow. On the other hand, the ultrasound echo used in this paper has high resolution, which may have enabled the renal arteries to be delineated and consequently renal blood flow to be calculated.

We investigated the effects of high intensity intermittent exercise (HIIE) and moderate intensity continuous exercise (MICE) on renal hemodynamics using ultrasound echo (Kawakami et al., 2024). As the authors point out, the HIIE protocol was set up to end with 2 minutes of low intensity exercise before measuring renal hemodynamics. This was done because stopping exercise immediately after high intensity exercise may be associated with a rapid decrease in venous perfusion. The high intensity of the HIIE protocol corresponds to the 140% lactate threshold (LT) intensity in our previous study, and exercise at 140% LT intensity reduced renal blood flow by approximately 50%, and the extent of this reduction was comparable to that immediately after intense exercise (Kawakami et al., 2018). The high intensity of the HIIE protocol appears to be sufficient (equivalent to a blood lactate level of 4 mmol/L) and the noradrenaline levels significantly increased immediately after HIIE and remained significantly higher in 60 min after exercise, it seems unlikely that our findings are more reflective of the rapid responsiveness of the renal vasculature to the cessation of low intensity exercise than to HIIE.

As renal hemodynamics are strongly influenced by exercise intensity and duration, decisions regarding exercise intensity and duration are significantly important. In the paper, the influences of HIIE or MICE on renal hemodynamics were examined after exercise, and intermittent short periods of HIIE were not detected to decrease renal blood flow or increase kidney injury risks (Kawakami et al., 2024). However, renal hemodynamic evaluation during exercise was technically difficult, so it was evaluated immediately after exercise. In the future, a more comprehensive understanding of the influence of exercise on renal hemodynamics may be achieved if it becomes possible to perform renal hemodynamic assessment during exercise.

## AUTHOR CONTRIBUTIONS

S.K. drafted manuscript; T.Y., S.K., A.I., K.F., T.M, S.N., K.M., Y.U., Y.H. and R.M. edited and revised manuscript; S.K., T.Y., S.K., A.I., K.F., T.M, S.N., K.M., Y.U., Y.H. and R.M. approved final version of manuscript.

## FUNDING INFORMATION

None.

## CONFLICT OF INTEREST STATEMENT

None.

## ETHICS STATEMENT

All procedures involving human participants were performed in accordance with the ethical standards of the institutional and/or national research committee at which the studies were conducted (Ethics Committee of Fukuoka University Approval No. 22‐06‐M1).
